# Supplementary material for: Geographic variation in the advertisement calls of Hyla eximia and its possible explanations
Source: PeerJ. 2014 Jun 17;2:e420. doi: 10.7717/peerj.420 (PMC4081300; doi:10.7717/peerj.420)
Supplement: Supplemental Information [file peerj-02-420-s001.doc]

**Supplementary Information**

Supplementary Figure S1. Call attributes measured with Avisoft-SASLab Pro™.


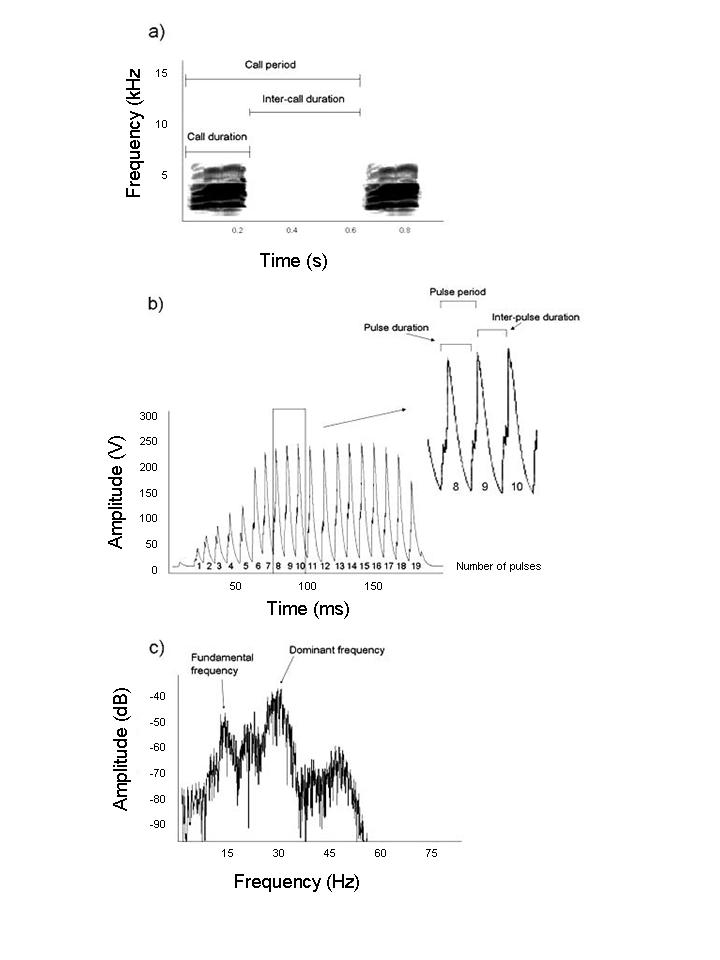


a) Spectrograms of *Hyla eximia* advertisement call.

b) Pulse train analysis. Highlighted are three pulses (8-10) to show pulse-specific properties.

c) Power spectrum (logarithmic) indicates the higher peak power (dominant frequency) and the basic harmonic (fundamental frequency) of the call.

Supplementary Table S1: Covariance analysis of call attributes of *Hyla eximia*, *Hyla arenicolor* and *Tlalocohyla smithii*.

|  | ***Hyla eximia*** | | | | | | | ***Hyla arenicolor*** | | ***Tlalocohyla smithii*** | | | | |
| --- | --- | --- | --- | --- | --- | --- | --- | --- | --- | --- | --- | --- | --- | --- |
|  | CD | IC | NP | PD | PA | DF | FF | NP | PA | CD | IC | NP | PD | DF |
| **T (°C)**  **F_(1,0.05)_**  **P** | 30.245  <0.001 | 10.069  0.001 | 2.714  0.101 | 3.651 0.058 | 0.003  0.950 | 7.799  0.006 | 2.934  0.089 | 5.884 0.059 | 18.943  0.012 | 0.703 0.416 | 0.164 0.692 | 8.126 0.012 | 1.077 0.318 | 9.792  0.007 |
| **CC**  **F_(2,0.05)_**  **P** | 82.381  <0.001 | 20.973  <0.001 | 189.87  <0.001 | 62.814  <0.001 | 0.206  0.814 | 11.609  <0.001 | 3.279 0.040 | 6.831 0.047 | 8.574 0.042 | 5.579 0.017 | 3.208  0.076 | 7.380 0.006 | 6.646 0.01 | 0.925 0.419 |
| **TXCC**  **F_(2,0.05)_**  **P** | 1.264  0.285 | 0.947  0.390 | 0.530 0.589 | 3.041  0.051 | 0.001  0.998 | 2.511 0.085 | 5.427 0.005 | 2.880 0.150 | 10.183 0.033 | 1.152 0.346 | 8.325  0.013 | 9.618 0.002 | 0.003 0.955 | 2.735 0.099 |

Notes:

T (°C) = Corporal temperature; CC = chorus composition; P = P value. The third main row shows the interaction between body temperature and chorus composition.

Supplementary Song description S1: Description of the advertisement calls of *H. eximia*, *H. arenicolor* and *Tlalocohyla smithii*.

The advertisement call of *Hyla eximia* consists of single note made of many pulses and reproduced several times (see below). It lasts less than a quarter of a second (mean ± SD = 0.207 ± 0.029 s), has a highly variable inter-call duration (range 0.24-1.51 s) and a call period of 0.676 (± 0.244) s. The call is made of 18.27 ± 3.919 pulses, each of which lasts 0.005 ± 0.001 s, so the pulses within a note are emitted at quick intervals (0.0114 ± 0.0041 s), and with a pulse period of 0.017 ± 0.005 s (pulse rate = 84.92 ± 22.04 Hz). Pulses are produced an amplitude of 0.23 ± 0.157 V, with a pulse peak frequency of 2.435 ± 0.321 kHz. The dominant frequency of the call is 2.645 ± 0.370 kHz, and its fundamental frequency is 0.627 ± 0.274 kHz.

The advertisement call of *Hyla arenicolor* is roughly three times longer (0.884 ± 0.182 s) than the typical call of *H. eximia* and is delivered at a somewhat slower rate (inter-call duration = 1.59 ± 0.473 s). It is made of only a few more pulses than the calls of *H. eximia*, but it pulse duration (0.008 ± 0.003 s), inter-pulse duration (0.04 ± 0.007 s) and amplitude (0.37 ± 0.21 V) are larger, whereas the pulse rate is much smaller (24.83 ± 4.91 Hz). Calls of *H. arenicolor* are lower-pitched than those of *H. eximia* (dominant frequency = 1.265 ± 0.666 kHz, pulse peak frequency =1.467 ± 0.66 kHz). We could not discern a fundamental frequency in our recordings of *Hyla arenicolor*.

The structure of the call of male *Tlalocohyla smithii* is very different from those of *H. eximia* and *H. arenicolor*. Often the males emit calls with two elements; a long note followed by an extremely short one. Most individuals, however, produce more long than short notes, and in several records the later are missing. Some males produced a third note with different structure and duration than the previous two, but it is not clear whether this is also part of the advertisement call. For our analyses we considered just the first note, as it is the more constant element in our recordings, thus the more likely to interfere with the advertisement calls of *H. eximia* calls. Long notes are almost twice as long as the call of *H. eximia* (0.47 ± 0.06 s) and have a similar number of pulses as those of *H. arenicolor* (23.734 ± 4.935). They are short (pulse duration =0.0048 ± 0.0004 s; inter-pulse duration =0.0204 ± 0.0034 s) and have a low amplitude (0.2142 ± 0.1522 V), with a pulse rate of 48.22 (± 7.66). Frequency attributes of *T. smithii* advertisement calls are different from those of the other species; they have a high dominant frequency (4.45 ± 0.158), high pulse peak frequency (4.316 ± 0.163), and high fundamental frequency (1.915 ± 1.514).
