# Supplementary material for: Geographic variation in the advertisement calls of Hyla eximia and its possible explanations
Source: PeerJ. 2014 Jun 17;2:e420. doi: 10.7717/peerj.420 (PMC4081300; doi:10.7717/peerj.420)
Supplement: Table S2 [file peerj-02-420-s002.doc]

Supplementary Table S2. GenBank access information of *Hyla eximia* sequences.

| ***Individual*** | ***Population*** | ***Latitude*** | ***Longitude*** | ***mtDNA***  ***cyt-b*** | ***mtDNA***  ***ATPase*** | ***nDNA***  ***Rho*** | ***nDNA***  ***POMC*** |
| --- | --- | --- | --- | --- | --- | --- | --- |
| **E_AG-01** | San Agustín, Jalisco. | 20° 17’ 53.15’’ N | -102° 37’ 47.7’’ W |  | KJ885579 |  |  |
| **E_AG-04** | San Agustín, Jalisco. | 20° 17’ 53.15’’ N | -102° 37’ 47.7’’ W | KJ719624 | KJ885580 | KJ611958 | KJ611979 |
| **E_AG-14** | San Agustín, Jalisco. | 20° 17’ 53.15’’ N | -102° 37’ 47.7’’ W | KJ719625 | KJ885581 | KJ611959 | KJ611980 |
| **E_K58-17** | Km 58, San Luis Potosí. | 22° 01’ 12.18’’ N | -100° 36’ 7.26’’ W | KJ719626 | KJ885582 | KJ611961 | KJ611981 |
| **E_K58-18** | Km 58, San Luis Potosí. | 22° 01’ 12.18’’ N | -100° 36’ 7.26’’ W | KJ719627 | KJ885583 | KJ611962 | KJ611982 |
| **E_K58-20** | Km 58, San Luis Potosí. | 22° 01’ 12.18’’ N | -100° 36’ 7.26’’ W | KJ719628 |  | KJ611963 | KJ611983 |
| **E_L-09** | Laguna de Gerardo, San Luis Potosí. | 22° 38’ 27.36’’ N | -100° 26’ 20.94’’ W | KJ719629 |  |  |  |
| **E_L-19** | Laguna de Gerardo, San Luis Potosí. | 22° 38’ 27.36’’ N | -100° 26’ 20.94’’ W |  |  |  | KJ611984 |
| **E_L-20** | Laguna de Gerardo, San Luis Potosí. | 22° 38’ 27.36’’ N | -100° 26’ 20.94’’ W |  | KJ885584 | KJ611964 | KJ611985 |
| **E_LM-G3** | Lagos de Moreno, Jalisco. | 21° 21’48.59’’ N | -101° 55’ 44.77’’ W | KJ719630 | KJ885585 |  |  |
| **E_LM-G4** | Lagos de Moreno, Jalisco. | 21° 21’48.59’’ N | -101° 55’ 44.77’’ W | KJ719631 | KJ885586 |  |  |
| **E_MA-01** | Magdalena, Jalisco. | 20° 54’ 0.41’’ N | -103° 59’ 36.85’’ W |  |  |  | KJ611986 |
| **E_MA-02** | Magdalena, Jalisco. | 20° 54’ 0.41’’ N | -103° 59’ 36.85’’ W | KJ719632 | KJ885587 | KJ611965 |  |
| **E_MA-03** | Magdalena, Jalisco. | 20° 54’ 0.41’’ N | -103° 59’ 36.85’’ W | KJ719633 | KJ885588 |  | KJ611987 |
| **E_MA-15** | Magdalena, Jalisco. | 20° 54’ 0.41’’ N | -103° 59’ 36.85’’ W |  |  |  | KJ611988 |
| **E_PM-01** | Presa de Malinaltenango, Edo. de México. | 18° 48’ 32.01’’ N | -99° 43’ 26.18’’ W | KJ719634 | KJ885589 | KJ611966 | KJ611989 |
| **E_PM-04** | Presa de Malinaltenango, Edo. de México. | 18° 48’ 32.01’’ N | -99° 43’ 26.18’’ W | KJ719635 | KJ885590 |  | KJ611990 |
| **E_PM-14** | Presa de Malinaltenango, Edo. de México. | 18° 48’ 32.01’’ N | -99° 43’ 26.18’’ W |  | KJ885591 | KJ611967 |  |
| **E_R-10** | El Realejo, San Luis Potosí. | 22° 40’ 23.98’’ N | -100° 25’ 5.45’’ W |  |  |  | KJ611991 |
| **E_R-12** | El Realejo, San Luis Potosí. | 22° 40’ 23.98’’ N | -100° 25’ 5.45’’ W | KJ719636 | KJ885592 |  |  |
| **E_R-16** | El Realejo, San Luis Potosí. | 22° 40’ 23.98’’ N | -100° 25’ 5.45’’ W | KJ719637 | KJ885593 | KJ611968 | KJ611992 |
| **E_SA-01** | El Realejo, San Luis Potosí. | 22° 40’ 23.98’’ N | -100° 25’ 5.45’’ W | KJ719638 | KJ885594 |  |  |
| **E_SA-04** | Sierra de Álvarez, San Luis Potosí. | 22° 01’ 52.56’’ N | -100° 36’ 49.86’’ W | KJ719639 | KJ885595 | KJ611969 |  |
| **E_SA-20** | Sierra de Álvarez, San Luis Potosí. | 22° 01’ 52.56’’ N | -100° 36’ 49.86’’ W |  | KJ885596 | KJ611970 | KJ611993 |
| **E_SE-05** | Rancho Santa Elena, Hidalgo. | 20° 08’ 7.38’’ N | -98° 30’ 43.50’’ W | KJ719640 | KJ885597 | KJ611971 | KJ611994 |
| **E_SE-14** | Rancho Santa Elena, Hidalgo. | 20° 08’ 7.38’’ N | -98° 30’ 43.50’’ W | KJ719641 | KJ885598 |  |  |
| **E_SE-18** | Rancho Santa Elena, Hidalgo. | 20° 08’ 7.38’’ N | -98° 30’ 43.50’’ W | KJ719642 | KJ885599 | KJ611972 |  |
| **E_SP-01** | San Pedro Tlaltizapán, Edo. de México. | 19° 11’ 41’’ N | -99° 30’ 13’’ W | KJ719643 | KJ885600 | KJ611973 | KJ611995 |
| **E_SP-03** | San Pedro Tlaltizapán, Edo. de México. | 19° 11’ 41’’ N | -99° 30’ 13’’ W | KJ719644 | KJ885601 |  | KJ611996 |
| **E_SP-05** | San Pedro Tlaltizapán, Edo. de México. | 19° 11’ 41’’ N | -99° 30’ 13’’ W | KJ719645 |  |  |  |
| **E_SP-06** | San Pedro Tlaltizapán, Edo. de México. | 19° 11’ 41’’ N | -99° 30’ 13’’ W |  |  | KJ611974 | KJ611997 |
| **E_SR-15** | Playita de San Rafael, Jalisco. | 20° 02’ 52.98’’ N | -103° 09’ 46.26’’ W | KJ719646 | KJ885602 |  | KJ611998 |
| **E_SR-16** | Playita de San Rafael, Jalisco. | 20° 02’ 52.98’’ N | -103° 09’ 46.26’’ W | KJ719647 |  | KJ611975 | KJ611999 |
| **E_SR-18** | Playita de San Rafael, Jalisco. | 20° 02’ 52.98’’ N | -103° 09’ 46.26’’ W | KJ719648 | KJ885603 | KJ611976 | KJ612000 |
| **E_VJ-02** | Valle de Juárez, Jalisco. | 19° 57’ 20.94’’ N | -102° 56’ 27.78’’ W | KJ719649 | KJ885604 |  | KJ612001 |
| **E_VJ-03** | Valle de Juárez, Jalisco. | 19° 57’ 20.94’’ N | -102° 56’ 27.78’’ W | KJ719650 |  | KJ611977 | KJ612002 |
| **E_VJ-07** | Valle de Juárez, Jalisco. | 19° 57’ 20.94’’ N | -102° 56’ 27.78’’ W | KJ719651 |  | KJ611978 | KJ612003 |
